# Do Ultrasound Lung Abnormalities Correlate to Biomarkers and Male Gender in Rheumatoid Arthritis Patients? A Monocentric Cross-Sectional Study

**DOI:** 10.3390/jcm13123534

**Published:** 2024-06-17

**Authors:** Francesca Bandinelli, Maurizio Benucci, Ilenia Mallia, Ilaria Mauro, Nikita Pecani, Francesca Li Gobbi, Mariangela Manfredi, Serena Guiducci, Barbara Lari, Valentina Grossi, Maria Infantino, Gianfranco Giannasi

**Affiliations:** 1 Rheumatology Department, San Giovanni di Dio Hospital, Usl Tuscany Center, 50143 Florence, Italy; maurizio.benucci@uslcentro.toscana.it (M.B.);; 2 Rheumatology Division, Department of Experimental and Clinical Medicine, University of Florence, 50141 Florence, Italy; 3 Immunology and Allergology Laboratory Unit, San Giovanni di Dio Hospital, Usl Tuscany Center, 50143 Florence, Italy; mariangela.manfredi@uslcentro.toscana.it (M.M.); v.grossi@uslcentro.toscana.it (V.G.); m.infantino@uslcentro.toscana.it (M.I.); 4 Emergency Department, San Giovanni di Dio Hospital, Usl Tuscany Center, 50143 Florence, Italy; gianfranco.giannasi@uslcentro.toscana.it

**Keywords:** lung ultrasound, rheumatoid arthritis, interstitial lung disease

## Abstract

**Background**: Lung ultrasound (LUS) is a tool of growing interest in Rheumatoid Arthritis (RA) oligo- symptomatic ILD to avoid. **Objective**: We aimed to evaluate (i) the prevalence of pleural (PLUS) and parenchymal (PAUS) abnormalities in LUS in the RA population and their possible correlation to biomarkers; (ii) the predictivity of gender, smoking habits, previous infections (past COVID-19 tuberculosis), and treatments; (iii) the differences in LUS between sexes. **Methods**: We collected the data of 155 (15 early and 140 late) RA patients with mild respiratory symptoms, evaluating PLUS and PAUS, in fourteen lung areas and also summing the scores (LUS-T). **Results**: Only 13/155 (8.4%) were completely negative; LUS correlated to age (all parameters *p* 0.0001), rheumatoid factor IgM (PLUS *p* 0.0006, PAUS *p* 0.02, LUS-T *p* 0.001) and ACPA (*p* 0.001, 0.006, 0.001, respectively), and PLUS also correlated to IL6 (*p* 0.02). The male gender was predictive of all LUS evaluations (*p* 0.001, 0.05, 0.001, respectively), which were higher than in women (*p* 0.001, 0.01, 0.001, respectively). Other potential risk factors were independent, except biological treatments, which showed a low predictivity to PLUS (*p* < 0.05). **Conclusions:** We can conclude that LUS is a useful technique in RA low respiratory symptoms and correlates with age, the most important RA biomarkers, and male sex.

## 1. Introduction

Rheumatoid arthritis (RA) is an inflammatory syndrome, characterized by definite genetic [1,2] and environmental risk [3,4]. It can lead to bone destruction and handicap.

RA interstitial lung disease (ILD) is a one of the most frequent systemic extra-articular complications and represents the second cause of mortality, after cardiovascular diseases [5,6]; it is associated with poor prognosis and high hazard of developing acute exacerbations and infections [6].

RA-ILD often precedes the onset of RA [7], and most subjects have initial subclinical airway abnormalities [8] that successively can evolve into severe lung involvement, with a mean survival range of three to ten years; for approximately 36% of cases, this range is shortened to the first five years of disease diagnosis [9].

To understand the types of lung involvement that can be present in RA, differences should be considered with regard to anatomic compartments of the lung, including airways, parenchyma, pulmonary vasculature, and pleura [8]. Each of these pulmonary regions have unique anatomic and functional abnormalities associated with RA characteristics: for instance, small or large airways are initially pathologically involved with bronchial inflammation evolving into thickening, bronchiectasis, bronchiolitis, or mosaic air trapping. While the prevalence of parenchymal lung disease in RA varies from 7 to 79% in different studies, depending on the heterogeneity of demographic and outcome measures employed to estimate it, recent data highlighted that pleural disease might be commonly identified in up to 50% of imaging in RA [8] and have an important impact on lung elasticity and respiratory functions, with pathologically different stages ranging from mild thickening to severe inflammation with nodularity and consolidation [10,11,12].

For this reason, the early diagnosis of ILD is useful to manage tailored and appropriate health interventions that can prevent successive spreads and reduce morbidity and mortality [13]. Its early identification represents an opportunity to avoid ILD progression, improve long-term outcomes and treat patients promptly with antifibrotic treatments, when indicated [13,14]. Furthermore, with most patients being oligo-symptomatic despite significant radiological abnormalities [15], optimal tools for early diagnosis and periodic screening are deemed essential during the follow-up, in the presence of predisposal conditions [16,17,18].

High-resolution chest computerized tomography (HRCT), more sensitive than traditional X-rays, might highlight early signs (subpleural fibrotic nodules and septa, traction bronchiectasis, reticulation, and micro-honeycombing) of usual interstitial pneumoniae (UIP), the most common pattern of RA-ILD [3,19,20].

Though low-dose chest computerized tomography (CT) was recently introduced to screen high-risk populations for tuberculosis and COVID-19 pneumonia [21,22], the radiation exposure for patients still represents a daily obstacle for timely detection of RA-ILD [23,24].

In this context, lung ultrasound (LUS), considered in the past a “forbidden zone”, is a new, relatively safe, rapid-use tool of growing interest in clinical practice that recently moved from the research frontiers of Systemic Sclerosis (Ssc) [25,26,27] to COVID-19 pneumonia [28] and pediatric patient [29] evaluation.

Even if air is not a favorable medium for transmission of LUS waves, the identification of vertical comet artifacts (B lines, due to fluid and collagen accumulation) and pleura layer morphological abnormalities has shown good sensitivity and concordance with the extent and severity of ILD in HRCT in autoimmune diseases and RA [30,31,32,33].

In the past decades, several RA features (sex, age, clinical and serological variables, and drug pathways) have been proposed as potential risk factors for ILD [34], and various circulating biomarkers have been linked to ILD survival [35]. However, an international consensus has not yet been reached, and their utility in early diagnosis of RA-ILD is still unclear.

Independent predictors of worsening RA-ILD were reported to be the usual interstitial pneumonia (UIL) pattern, low restrictive parameters of respiratory tests, cigarette smoking, and higher anticitrullinated protein antibody titers (ACPA) [13,36], while there has been a long-running debate whether methotrexate (MTX) can cause ILD, as it can lead to inflammatory pneumonitis but not to a truly fibrotic progressive disease [37].

Otherwise, this historic inability to discriminate between RA-ILD and lung “toxicity” was recently revised in the literature [37], suggesting that MTX is not correlated to ILD and might indeed slow its progression [38].

While limited evidence based only on cross-sectional or retrospective studies showed that tumor necrosis factor inhibitors (anti-TNF-alpha) might be associated with a potential mild increase in lung impairment, other non-anti-TNF-alpha biologics (rituximab, abatacept, and tocilizumab) seem to be safe for RA-ILD [39] and might be associated with lower short-term progression of ILD [36].

With this background, the aims of the present study were to evaluate (i) the prevalence of pleural (PLUS) and parenchymal (PAUS) abnormalities in LUS in the RA population and their possible correlation to clinical and serological biomarkers; (ii) the predictivity of gender, smoking habits, previous COVID-19 hospitalization or latent tuberculosis treatments for LUS; and iii) the differences in LUS scores between sexes.

## 2. Materials and Methods

### 2.1. Study Design

We conducted a cross-sectional and retrospective analysis of RA patients, encountered in routine clinical practice, presenting mild respiratory complications of RA disease (slight shortness of breath when hurrying on level ground or walking up a slight hill). These patients were initially referred to the early arthritis outpatient clinic by general practitioners or followed at a second-level RA outpatient clinic for established disease, at San Giovanni di Dio Hospital in Florence (Italy), following regional flowchart guidelines, during the period of 7 July to 11 December 2023. In this study, we delineated respiratory symptoms in patients with dyspnea using the Medical Research Council Questionnaire (m-MRC) self-rating tool to measure breathlessness during day-to-day activities on a scale from 0 to 4: 0, no breathlessness except on strenuous exercise; 1, shortness of breath when hurrying on level ground or walking up a slight hill; 2, walks slower than people of same age on level ground because of breathlessness or has to stop to catch breath when walking at their own pace on level ground; 3, stops to catch breath after walking ~100 m or after few minutes on level ground; and 4, too breathless to leave the house or breathless when dressing or undressing [40].

Routine LUS was performed for patients at the Emergency Department of San Giovanni di Dio Hospital (Usl Tuscany Center, Florence, Italy), to assess possible parenchymal and pleural abnormalities, following Italian Health Ministry legislation (22 April 2021, No. 52), with a protocol defined for other interstitial respiratory diseases [41].

### 2.2. Participants

Data from a total of 155 consecutive patients with RA aged 18 years and older were retrospectively gathered through the Argos electronic system of Usl Tuscany Center. Data were collected for those patients who exhibited oligo-symptomatic respiratory symptoms (m-MRC 1 and 2) at clinical examination, not correlated to recent infective respiratory diseases and not previously treated with antifibrotic drugs for ILD.

All early RA patients (with arthritis onset less than one year before LUS evaluation) diagnosed under the EULAR/ACR 2010 criteria [42] were followed up for almost six months to confirm established disease. Privacy consent for anonymous analysis and publication of routine clinical data was given by each patient and saved in the Argos electronic chart of Usl Tuscany center, as per the Declaration of Helsinki on investigation of humans and according to Tuscany Region Institutional Review Board resolution (No. 450) and Italian legislation (authorization No. 9, 12 December 2013).

Exclusion criteria encompassed pregnancy, concomitant infections and tumors, neurological disorders (such as multiple sclerosis and cognitive impairment), or known serious respiratory disorders (severe COPD or asthma, cystic fibrosis, sarcoidosis). Smoking (present, past, and high passive exposure) was not considered an exclusion parameter, being an important trigger pathogenetic factor for RA and ILD [3]. Patients with previous latent tuberculosis (positive QuantiFERON test for tuberculosis mycobacterium, usually performed in all RA patients in clinical practice) were routinary evaluated by infective disease specialists to exclude active disease. Patients with past COVID-19 infection (almost six months previous than evaluation) were not excluded, being common in the RA population and pandemic period; patients that necessitated domiciliary assistance or hospitalization were classified using the World Health Organization (WHO, 0–10) severity score of COVID-19 infection [43].

### 2.3. Measurements

We collected demographic, biochemical, and clinical attributes of participants, analyzing face-to-face interviews, clinical evaluations, and patient records.

Clinical data on disease duration, disease activity (DAS28 ESR and DSAS28 CRP performed contextually for clinical examination by expert rheumatologists [MB, FB, FLG]) and treatment (MTX, jak inhibitors, biological anti-TNF-alpha, and biological non-anti-TNF [tocilizumab, sarilumab, rituximab, abatacept]) were collected from the medical records.

Furthermore, in patients with m-MRC higher than 2, ILD was evaluated through HRCT (saved in the Elephant.net v. 2.85.00 electronic system of Usl Tuscany Center), analyzing elementary lesions for subpleural fibrotic nodules, traction bronchiectasis, ground glass, reticulation, and honeycombing. Routine HRCT, which can exceed the radiation dose of chest radiography by 100 times, was not performed in m-MRC 1, due to stochastic cancer risk from high radiation exposure [14,23].

In the presence of radiological mild–severe interstitial lung disease (ILD), the severity and classification of lung function tests were defined as normal: diffusing capacity of the lungs for carbon monoxide (DLCO) > 75% of predicted, up to 140%; mild: 60% to LLN (lower limit of normal); moderate: 40 to 60%; severe: <40% [44]. DLCO < 60% or DLCO < 75% associated with a reduction of forced vital capacity [FVC] <80% of that predicted were considered significant as restrictive parameters for pulmonary function rate (PFR) tests [44].

The following blood tests were analyzed at the Clinical Pathology and Immunology-Allergy Laboratory of Usl Tuscany Centre (Florence, Italy) using venous blood specimens collection after 12 h overnight fasting: Anti-Citrullinated Peptide Antibodies (U/mL, ACPA, Thermo Fisher, Uppsala, Sweden), Rheumatoid Factor IgM (U/mL, RF, Siemens AG, Munich, Germany), IgA and IgG (U/mL, RF, Thermo Fisher, Uppsala, Sweden), erythrocyte sedimentation rate (mm/h, ESR, Alifax, Padoa, Italy), C-Reactive Protein (mg/dL, CRP, Beckman Coulter Inc, Brea, CA, USA), interleukin 6 (pg/mL, IL6, Invitrogen, Bender MedSystem GmbH, Vienna, Austria), and complement functional activity for the determination of classical, alternative, and mannitol binding lectin (MBL) pathways (percentage, Euro diagnostica AB, Malmö, Sweden).

Additionally, based on clinical symptoms associated with arthritis (e.g., Raynaud or sicca syndrome, muscle pain and weakness), we explored the association with other connective manifestations, through investigation with specific blots for Anti-Cellular Antibodies (ANAs) tested by indirect immunofluorescence assay using HEp-2 cells (Euroimmun, Lübeck, Germany) according to ICAP classification [45], when indicated.

An expert senior ultra-sonographer (GG), director of the Emergency Unit of San Giovanni di Dio Hospital, consecutively studied, with LUS, 155 RA outpatients and trained, during the six months of examinations, three junior residents [IMal, IMau, NP], unexperienced in LUS, from the Rheumatology specialization school of the University of Florence.

A standard sequence of LUS evaluation of fourteen (3 posterior, 2 lateral, and 2 anterior) intercostal spaces was performed using landmarks of chest anatomic lines, with progressive numbering starting from the right posterior basal regions, belong the protocol of Soldati et al. [28], using a Samsung RS85 Prestige machine with a linear 3–12 MHz array (L3–12A) probe.

LUS abnormalities, based on right and left evaluation, were defined using the recent criteria provided by the OMERACT taskforce for LUS in SSc [27].

Furthermore, pleural (PLUS) and parenchymal (PAUS) abnormalities, termed using the Fisher definition [46], were semi-quantitatively evaluated as 0 (negative) to 3, with separated scores, with a final total score (LUS-T) as the sum of single parameters.

LUS-T zero was defined as a thin, regular, and continued pleural line and the absence of B lines in all scanning sites.

LUS abnormalities were scored as follows:

- PLUS 1, non-linear and non-homogeneous, thickened pleural line;
- PLUS 2, disrupted pleural line (“fragmented”);
- PLUS 3, subpleural consolidation (subpleural echo-poor region or “tissue-like”);
- PAUS 1, discrete divergent B lines;
- PAUS 2, confluent B lines;
- PAUS 3, dense confluent areas (“whiteout”) that persist during the respiratory cycle.

At the end of the procedure, the clinician wrote for each quadrant the score obtained, and the images were stored in the US machine. To estimate the US intra-reader agreement, the saved images of patients were read almost two months after the initial scanning by the same expert ultra-sonographer (GG) who performed the first examination, unaware of the previous results. For the inter-reader agreement, the junior residents’ results were compared with those of the senior resident, whose results were considered the gold standard, to verify their learning objectives and progress.

### 2.4. Statistical Analysis

The sample size was calculated according to the methodology suggested in recent studies on ILD in RA [32,47]: 155 patients in the studied population achieved >80% statistical power, with an α level of 0.05 and beta 0.2.

The Kolmogorov–Smirnov and Shapiro–Wilk tests were used to evaluate the distribution of variables. Data with non-normal distribution were assessed using Spearman correlation and Mann–Whitney tests. Categorical data were compared among groups using the Chi-square test. Descriptive statistics were expressed as median and interquartile range (IQR) for continuous parameters and percentage for categorical variables, as appropriate.

Linear regression was used to evaluate the predictivity of LUS scores with respect to gender, smoking habits, and use of methotrexate (present or past).

For intra-reader and inter-reader reliability for LUS, we adopted an intraclass correlation coefficient (ICC) for semi-quantitative assessment of both PLUS and PAUS on saved, mixed, and blind images, re-scored almost two months after the first examination. An ICC below 0.50 was considered poor; between 0.50 and 0.75, moderate; between 0.75 and 0.90, good; and above 0.90, excellent.

The level of statistical significance was set at a *p* value ≤ 0.05. All statistical analyses were performed using GraphPad prism 8.0. The reporting of this study conforms to STROBE guidelines [48].

## 3. Results

A total of 155 RA (15 early and 140 established arthritis) patients with mild respiratory symptoms at clinical examination, encountered during routine clinical practice, at early arthritis and second-level RA outpatient clinics of Usl Tuscany Center San Giovanni di Dio Hospital, underwent LUS examination at the Emergency Unit of San Giovanni di Dio Hospital from 7 July 2023 to 11 December 2023. The intra-reader score [GG] (IC > 0.9) was excellent for both LUS parameters examined. The inter-reader agreement of the senior resident (golden standard) in comparison to junior residents, after a six-month training period, was good (IC > 0.8) and excellent (IC > 0.9), as shown Figures S1 and S2 (Supplementary Materials).

**Figure 1:**
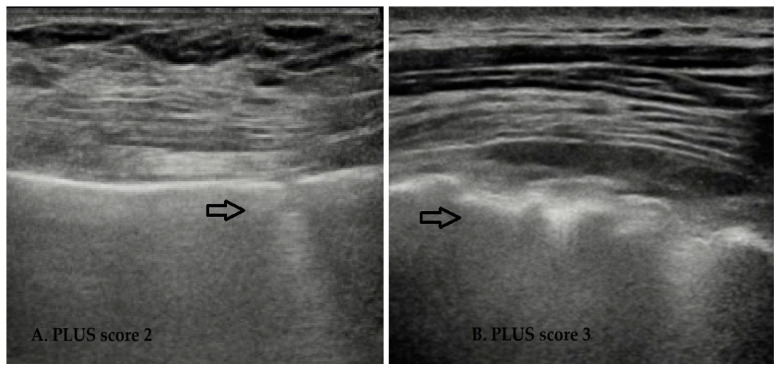
Pleural fragmentation (score 2, from 0 to 3, of pleural abnormalities) (arrow (**A**)) and consolidation (score 3, from 0 to 3, of pleural abnormalities) (arrow (**B**)) in RA patients upon lung ultrasound examination.

Only 13/155 (8.4%) patients did not present abnormalities in LUS, with the same percentage for PLUS (13/155, 8.4%), while patients with normal PAUS were more likely to present abnormalities (77/155, 49.6%). Pleural fragmentation (PLUS 2) and consolidation (PLUS 3) are shown in Figure 1. Pleural effusion was not visible in all patients in LUS.

A total of 32/155 (20.6%) patients, presenting m-MRC > 2, underwent HRCT with ILD diagnosis; otherwise, low DLCO reduction in respiratory tests was found only in five patients (<60% or a DLCO < 75% associated with a reduction in FVC < 80% of that predicted).

Only 9/155 (5.8%) patients had overlap with mild secondary connective disease (seven with Sjogren and two with antiphospholipid autoantibody positivity confirmed in two repeated withdrawals).

Only seven patients had between 2021 and 2022 a confirmed SARS-CoV-2 infection with symptoms that required assistance: two had domiciliary assistance (WHO 3), one was hospitalized without the need for oxygen therapy (WHO 4), one was treated with low-flow oxygen therapy (WHO 5), and three managed with non-invasive ventilation (NIV) (WHO 6).

### 3.1. Correlation of LUS Parameters with Demographic, Clinical, and Laboratory Biomarkers: Predictivity of Risks Factors for LUS Score

Demographic, clinical, and laboratory biomarker data and their possible correlation with LUS are shown in Table 1.

A significant correlation was found between PLUS, PAUS, and LUS-T scores and age (all *p* < 0.0001), but not duration and activity of disease, with no difference in LUS parameters between early and late RA.

Possible predictive factors, such as smoking habits, previous and concomitant methotrexate and anti-jak use, previous COVID-19 hospitalization, and latent tuberculosis were independent of LUS alterations. Male gender was significantly predictive of PLUS, PAUS, and LUS-T scores (*p* 0.001 and r 0.06, *p* 0.05 and r 0.02, *p* 0.001 and r 0.06, respectively); biological drugs were predictive only of PLUS and LUS- T (anti-TNF-alpha, *p* 0.04 and r 0.03, *p* 0.03 and r 0.03, respectively; non-anti-TNF-alpha, *p* 0.01 and r 0.04, *p* 0.01 and r 0.04, respectively). Between RA treated with abatacept and rituximab, only 14 patients naïve from previous anti-TNF-alpha or anti-IL6 (tocilizumab and sarilumab), presented predictivity of PLUS and LUS-T, even if less significantly (*p* 0.04 and r 0.2, both).

While inflammatory reactant markers (ESR, CRP) and all subtypes of complements were not associated with LUS abnormalities, RF IgM and ACPA correlated to all LUS scores and IL6 only to PLUS and LUS-T, as shown in Table 1.

### 3.2. Difference in LUS between Genders

As we can see from Table 2, the risk factors for pulmonary involvement and the clinical and concomitant treatments are not significantly different between the two gender populations examined.

Between biomarkers, only rheumatoid factor IgA was higher in men (M) in comparison to women (F) (*p* 0.02).

**Figure 2:**
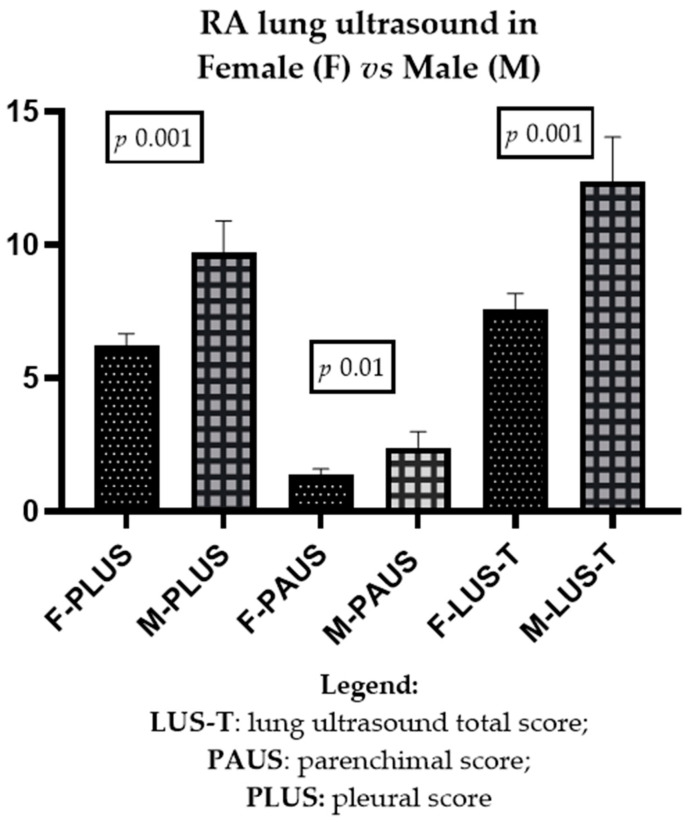
Differences, at ultrasound lung examination, between females and males in pleural (PLUS), parenchymal (PAUS), and total lung (LUS-T) scores.

Finally, as shown in Figure 2, at ultrasound lung examination, PLUS (*p* 0.001), PAUS (*p* 0.01) and LUS-T (*p* 0.001) were higher in men than in women, with analogous results for ILD abnormalities shown at HRCT (*p* 0.01) and DLCO reduction (*p* 0.001) (DLCO < 60% or DLCO < 75% associated with a reduction of FVC < 80% of that predicted).

The difference in ILD elementary abnormalities at HRCT between sexes, shown in Table S1 (Supplementary Materials), is not significant, and a definitive UIP pattern was present in a similar percentage for the two genders (19% F and 18.1% M). Otherwise, while subpleural fibrotic lines, nodules, and bronchiectasis were present in almost all patients, ground glass and reticulation were slightly more frequent in males (45% M vs. 19% F).

## 4. Discussion

Our cross-sectional study found parenchymal abnormalities in LUS in almost half of low symptomatic RA patients, similar to two recent studies by Santos Moreno and Otaola et al. that described the narrow base reverberation (B lines) in a high percentage in RA-ILD [33,49], also in the early phase of disease [33].

The B lines are considered sensitive and specific for screening collagen tissue deposit in interstitial subpleural interlobular septa in preclinical stages of ILD [25,27,50,51], but the mechanisms of their generation are still not fully understood and cannot discriminate the early cellular inflammation from the chronic fibrotic phase of ILD.

In this context, the diagnosis of pleural abnormalities is deemed essential [52], being a predominant early thoracic complication of RA that is easily recognized with LUS [47].

In our study, for the first time, pleural alterations were described in more than 90% of cases and confirmed by HRCT, showing subpleural fibrotic lines and nodules in almost all subjects with an m-MRC higher than two.

Although preliminary data on the diagnostic power of the fragmentation and thickening of pleural lines are promising and easily detected with a linear transducer [52], PLUS abnormalities are rarely specifically described in the literature and usually are scored together with B lines [27,28].

Recently, Moazedi et al. described B lines in 28%, subpleural nodes in 18%, and pleural fragmentation in 4% in LUS evaluation of RA patients totally asymptomatic for respiratory disorders, in accordance with HRCT results [47].

PLUS gives the opportunity to outline the typical RA thickening of parietal and visceral pleurae and the progressive fragmentation of the pleural line, which might assume an abnormal nodular appearance and lead to a mass-like pleural fibrosis with consequent lung restriction [10,11,12]. Furthermore, in pleural biopsy, the mesothelial cells are replaced by typical RA inflammatory epithelioid and multinucleated giant cells, sometimes combining in specific rheumatoid granulomas [11].

Despite extensive research in ILD on the underlying mechanisms responsible for the development of the disease, the interactions between lung fibroblasts and risk factors are multifaceted and remain sometimes controversial and poorly understood [53].

The potential predictors of RA–ILD, assessed in exploratory analysis and metanalysis, were older age (≥65 years old), RA biomarkers, activity of disease, smoking, and male sex [5,6].

In our study, all PLUS parameters correlated to age, ACPA, and RF, confirming previous data on ILD [18], but not to smoking habits, disease activity, and duration;, IL6 and male sex were related only to PLUS abnormalities.

Multiple issues demonstrated a high prevalence of ILD, also sub-clinically, in early RA and its key role in autoantibody production and RA progression [18]. On the other side, it has been reported that increased RF and ACPA antibody titers are predictors for the development of RA-ILD [18,54].

In fact, outside the synovial tissue, the citrullination pathways might be also present and upregulated in bronchoalveolar lavage cells and in lung fibroblasts of patients with RA-ILD [55].

In addition, combined traditional DMARDs and anti-TNF-alpha treatments are considered controversial hypothetic ILD risk factors [10,17], while abatacept and rituximab are considered preferable in the case of ILD [39].

In our results, the available anti-TNF-alpha and non-anti-TNF-alpha biological treatments seemed to be only modestly predictive of PLUS but not of PAUS, even if rituximab and abatacept could not be analyzed singularly due to the limited numbers included and the nature of the study, which did not allow a more precise evaluation during the study period.

On the other side, MTX and anti-jak drugs were ineffective for LUS, confirming the recent evidence that MTX does not increase the incidence or exacerbation of ILD and might improve survival [37,39,47].

Finally, the male gender is predictive of all LUS abnormalities and is associated with higher LUS values than females, with greater significance for PLUS. In fact, we know from the literature that pleural line alterations are quite common in males, especially in older age (>35 years) and with rheumatoid nodules [10]. We also demonstrated that males had higher RF-IgA levels, regarded as a predicting factor for poor prognosis of RA-ILD in recent studies [56], and showed poorer DLCO and HRCT involvement, without a significant difference in definitive UIP radiologic patterns in patients with ILD as demonstrated by HRCT. We suppose that LUS abnormalities present in patients with low initial respiratory symptoms might be predictive of future ILD in the male population of RA; however, for the limited number of patients studied in the male subpopulation, we can consider such findings as explorative data useful for future research.

As sex hormones are important risk or beneficial factors in the pathogenesis of autoimmune diseases and play a key role in maintaining lung homeostasis and facilitating tissue repair processes [57], previous animal models have suggested a protective effect of female sex hormones on pulmonary injury-induced inflammation and functional responses [58], showing that the lungs of knockout mice for estrogen receptor beta accumulated a higher quantity of collagen compared to wild-type controls [59] and, more recently, that the increased activation of estrogen alpha receptors mediates the male-predominant lung fibrosis, via the fibroblast microRNA transcriptome [60].

Otherwise, while males showed a higher abnormal HRCT and PFR rate, we cannot deduce the exact sensibility and specificity of this abnormality, because we performed these tests only in patients with m-MRC > 2, according to our guidelines and for ethical reasons regarding radiation exposure.

## 5. Conclusions

From our research, we might conclude that LUS offers an important opportunity to screen for RA-ILD, with rapid and inexpensive imaging techniques, to focus on severity elements overall in males and RF, ACPA, and IL6 higher risk populations, and to better select candidates for second-level successive HRCT in patients with low respiratory symptoms, reducing radiation exposure, which still represents a daily obstacle for timely RA-ILD detection.

Given also the low sensitivity of X-rays and pulmonary function tests in RA-ILD diagnosis, the detection, quantification, and description of lung changes suggestive of ILD in LUS in patients with RA might be important for other several reasons, with promising application: it might identify early changes in low symptomatic patients, useful to successively evaluate the potential prognostic value in terms of survival and to monitor lung disease progression and possible antifibrotic treatments.

Although thickening, fragmentation and consolidation of pleura are rarely studied, the diagnostic power of PLUS seems promising, and in our study, pleural abnormality description in RA patients was frequent.

Even more, LUS seemed to be a discriminative tool of the severity of ILD in males in comparison to females, together with RF IgA, even if we should judge these data only as initial explorative results.

Other future interventional clinical studies on larger numbers, in which participants are prospectively assigned, might evaluate the presence of PLUS abnormalities in early RA patients asymptomatic for respiratory disorders and their effects on biomedical or health-related outcomes, with comparison to HRCT and DLCO data.

## Figures and Tables

**Table 1:** Clinical, ultrasonography, and laboratory biomarkers of RA patient characteristics and their correlation and predictivity.

	RA Patients (*n*= 155)	Correlation of LUS and RA Biomarkers and Difference in LUS for Single Parameter Examined	*p*-Value
**Age** (median, IQR)	67 (56–76)	PLUS (*) **PAUS** **LUS-T**	**<0.0001** **<0.0001** **<0.0001**
Disease duration (median, IQR, N, %)	12 (5–20) years; early-RA (<1 year) (9.7%) vs. late RA	All LUS parameters	NS
LUS score (median, IQR)	PLUS: 6 (3–10) PAUS: 1 (0.5–2) LUS-T: 7 (4–12)	NA	NA
Smoking (N, %)	33 (26.4%)	All LUS parameters	NS
**Male gender (N, %)**	**30 (24%)**	PLUS (**) **PAUS** **LUS-T**	**0.001** **0.05** **0.001**
Connective disease overlap	9 (5.8%)	All LUS parameters	NS
DAS28(ESR) (median, IQR)	2.6 (2–3.4)	All LUS parameters	NS
DAS28 (CRP) (median, IQR)	2 (1.5–3.1)	All LUS parameters	NS
COVID-19 (WHO > 3) past infection and latent tuberculosis (N, %)	10 (8%)	All LUS parameters	NS
Previous and concomitant treatments			
Methotrexate and anti-jak (respectively)	99 (65.9%), 37 (29.6%)	All LUS parameters	NS
**Biologic anti-TNF-alpha and non-anti-TNF-alpha (respectively)**	**57 (45.6%), 68 (54.4%)**	PLUS (**) PAUS **LUS-T**	**0.04, 0.01** NS, NS **0.03, 0.01**
Laboratory tests			
CRP (mg/dL) (median, IQR)	0.21 (0.06–0.8)	All LUS parameters	NS
ESR (mm/h) (median, IQR)	17.5 (6–32.2)	All LUS parameters	NS
**RF IgM (U/mL) (median, IQR)**	73 (20–222)	PLUS (*) **PAUS** **LUS-T**	**0.0006** **0.02** **0.001**
**RF IgA (U/mL) (median, IQR)**	20 (6.3–43)	All LUS parameters	NS
**RF IgG (U/mL) (median, IQR)**	20 (8.9–29)	All LUS parameters	NS
**ACPA (U/mL) (median, IQR)**	129 (20–1600)	PLUS (*) **PAUS** **LUS-T**	**0.001** **0.006** **0.001**
**IL6 (pg/mL) (median, IQR)**	2.9 (2.9–9.6)	PLUS (*) PAUS **LUS-T**	**0.02** NS **0.02**
Classic, BML, alternative complement (median, IQR)	111 (87.4–123), 34 (9.8–71), 86.5 (67–96.7)	All LUS parameters	NS

Values were expressed in median and interquartile (IQR) with *p* of Spearman‘s r (*) and linear regression (**) tests; significant values are in bold; abbreviations: ACPA: anti-citrullinated protein antibodies, CRP: C-reactive protein, ESR: erythrocyte sedimentation rate, IL6: interleukin 6, LUS-T: lung ultrasound total score, NA: not applicable, NS: not significant, PAUS: parenchymal lung ultrasound score, PLUS: pleural lung ultrasound score, RF: rheumatoid factor, WHO: World Health Organization.

**Table 2:** Differences between genders in clinical, ultrasonography, lung involvement, biomarkers, and treatments of RA patients.

	Female (N = 125)	Male (N 30)	*p*-Value (*)
Age, years (median, IQR)	66.00 (53.5–76.00)	69 (63.5–75.00)	NS
Disease duration (median, IQR)	5 (12–20)	9 (3–15)	NS
DAS28 (ESR); DAS28 (CRP)	2.6 (2–3.4); 1.04 (1.5–3)	2.5 (1.7–3.4); 1.8 (1.5–3.2)	NS
LUS scores (median, IQR)	**PLUS: 5 (3–9)** **PAUS: 0.1 (0–2)** **LUS-T: 6 (3–10.2)**	**PLUS: 9.5 (5–13.25)** **PAUS: 1.5 (0.1–3)** **LUS-T: 11.5 (6–16.2)**	**0.001** **0.01** **0.001**
DAS28 (ESR); DAS28 (CRP) (median, IQR)	2.6 (2–3.4); 1.04 (1.5–3)	2.5 (1.7–3.4); 1.8 (1.5–3.2)	NS
Biomarkers			
**RF** IgM, **IgA**, IgG U/mL (median, IQR)	62 (20–182), **20 (5.3–39),** 20 (8.4–30) (*)	112 (20–457), **21 (18.5–151),** 20 (11–27.5)	NS IgM and IgG **0.02 IgA**
ACPA U/mL (median, IQR)	105. 5 (12.7–504.4)	239.5 (36.5–665.6)	NS
IL6 pG/mL (median, IQR)	2.9 (2.9–8.6)	4.4 (2.9–15.4)	NS
Lung involvement and factor of risk			
**HRCT ILD N (%)**	21 (16.8%) (**)	**11 (36.6%)**	**0.01**
**DLCO reduction (<60% or <75% with association of FVC reduction) N (%)**	2 (1.6%) (**)	**4 (13.3%)**	**0.001**
Smoking	22 (17.6%)	11 (36.6%)	NS
COVID-19 hospitalization and latent tuberculosis (treated with prophylaxis) N (%)	7 (5.6%)	4 (13%)	NS
Concomitant treatments			
Hydroxychloroquine, N (%)	17 (13.6%)	4 (13.3%)	NS
Methotrexate, N (%)	44 (35.2%)	12 (40%)	NS
Sulfasalazine, N (%)	1 (0.8%)	0 (0%)	NS
Leflunomide, N (%)	9 (7.2%)	1 (3.3%)	NS
Anti-TNF-alpha, N (%)	18 (35.2%)	4 (13.3%)	NS
Anti-jak inhibitors, N (%)	25 (20%)	5 (16.6%)	NS
Tocilizumab, N (%)	33 (26.4%)	10 (33.3%)	NS
Sarilumab, N (%)	7 (5.6%)	2 (6.6%)	NS
Rituximab, N (%)	3 (2.4%)	1 (3.3%)	NS
Abatacept, N (%)	33 (26.4%)	4 (13.3%)	NS

Values were expressed in median and interquartile (IQR) with *p* of Mann–Whitney (*) and percentage with *p* of Chi square (**) tests; significant values are in bold; abbreviations: ACPA: anti-citrullinated protein antibodies, CRP: C-reactive protein, ESR: erythrocyte sedimentation rate, FVC: forced vital capacity, HRCT: high-resolution chest tomography, ILD: interstitial lung disease, IL6: interleukin 6, LUS-T: lung ultrasound total score, NS: not significant, PAUS: parenchymal lung ultrasound score, PLUS: pleural lung ultrasound score, RF: rheumatoid factor, TNF: tumor necrosis factor.

## Data Availability

The data are available if required.

## Supplementary Materials

The following supporting information can be downloaded at: https://www.mdpi.com/article/10.3390/jcm13123534/s1: Figures S1 and S2: Intra-class correlation coefficient (ICC) of inter-reader agreement of parenchymal (PAUS) (S1) and pleural abnormality (PLUS) semiquantitative scores between senior (S) expert ultra-sonographer and three junior (J) rheumatology residents. Table S1: difference between sexes for HRCT elementary lesions in RA patients.
