# Supplementary material for: Do Ultrasound Lung Abnormalities Correlate to Biomarkers and Male Gender in Rheumatoid Arthritis Patients? A Monocentric Cross-Sectional Study
Source: J Clin Med. 2024 Jun 17;13(12):3534. doi: 10.3390/jcm13123534 (PMC11204435; doi:10.3390/jcm13123534)
Supplement: Supplementary file 1 [file jcm-13-03534-s001.zip › jcm-2993367-supplementary.pdf]

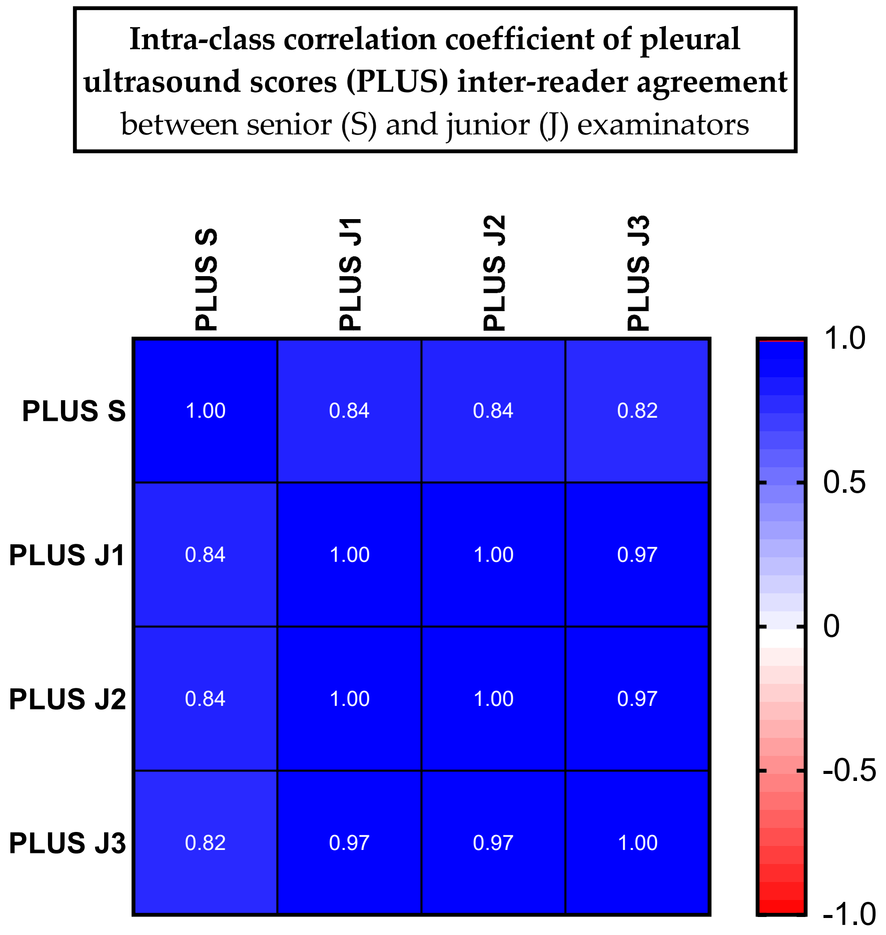

Figure S1. Intra-class correlation coefficient (ICC) of inter-reader agreement of pleural abnormalities (PLUS) semiquantitative scores between senior (S) expert ultra-sonographer and three junior (J) Rheumatology residents after six months of training in lung ultrasound. An ICC below 0.50 was considered poor, between 0.50 and 0.75, moderate, between 0.75 and 0.90, good, above 0.90, excellent.

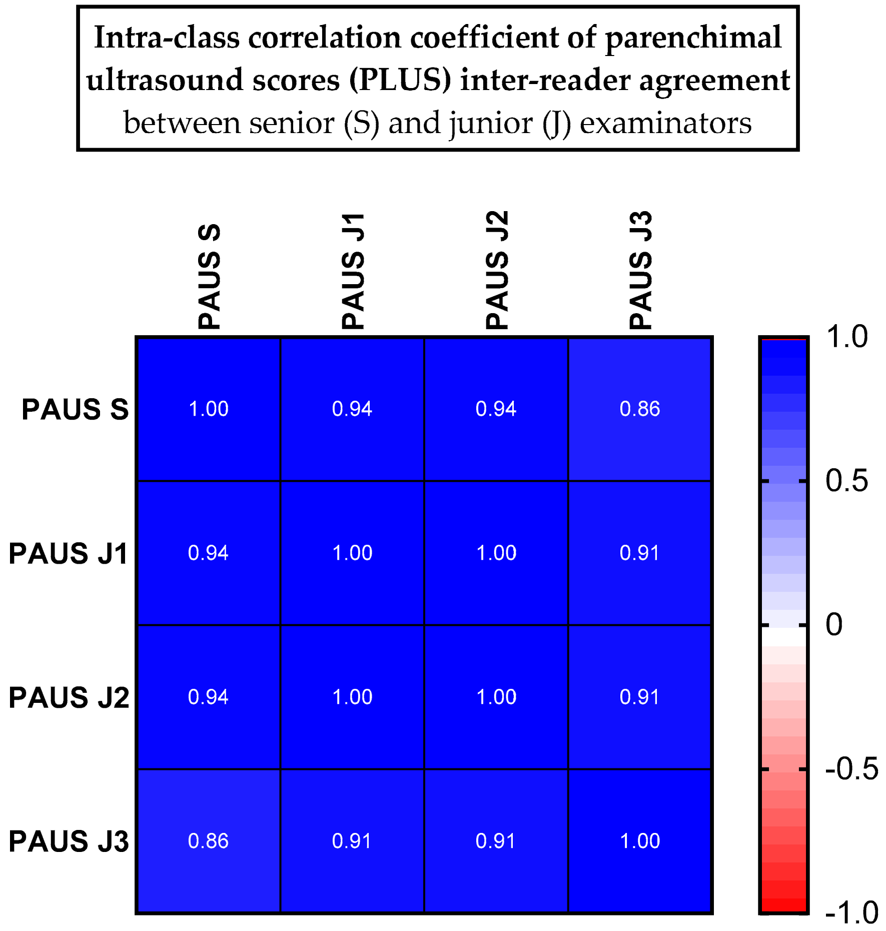

Figure S2. Intra-class correlation coefficient (ICC) of inter-reader agreement of parenchymal abnormalities (PAUS) semiquantitative scores between senior (S) expert ultra-sonographer and three junior (J) Rheumatology residents after six months of training in lung ultrasound. An ICC below 0.50 was considered poor, between 0.50 and 0.75, moderate, between 0.75 and 0.90, good, above 0.90, excellent.

**Table S1**	**Female**	**Male**	**p-value**
HRCT positive for ILD, N (%)	(N= 21)	(N=11)	
Subpleural fibrotic lines and nodules, N (%)	20 (95.2%)	11 (100%)	ns
Traction bronchiectasis, N (%)	15 (71.4%)	11 (100%)	ns
Reticulation, N (%)	3 (19.4%)	5 (45.4%)	ns
Ground glass, N (%)	4 (19%)	5 (45.4%)	ns
Honeycombing, N (%)	4 (19%)	2 (18.1%)	ns
UIP pattern established, N (%)	4 (19%)	2 (18.1%)	ns

Legend: Values are expressed in percentage with p of Chi square test; abbreviations: HRCT: High-resolution chest tomography, ILD: interstitial lung disease, ns: non-significant, RA: rheumatoid arthritis, UIP: usual interstitial pneumonia

Table S1. Difference between sexes of HRCT elementary lesions in RA patients
